# Perspective of Membrane Technology in Pomegranate Juice Processing: A Review

**DOI:** 10.3390/foods9070889

**Published:** 2020-07-07

**Authors:** Carmela Conidi, Enrico Drioli, Alfredo Cassano

**Affiliations:** 1 Institute on Membrane Technology, ITM-CNR, Via P. Bucci 17/C, 87036 Rende (CS), Italy; c.conidi@itm.cnr.it (C.C.); e.drioli@itm.cnr.it (E.D.); 2 Department of Engineering and of the Environment, University of Calabria, Via P. Bucci 45/A, 87036 Rende (CS), Italy; 3 State Key Laboratory of Materials-Oriented Chemical Engineering, College of Chemical Engineering, Nanjing Tech University, Nanjing 210009, China; 4 Center of Excellence in Desalination Technology, King Abdulaziz University (KAU-CEDT), Jeddah 21589, Saudi Arabia

**Keywords:** pomegranate, clarification, concentration, pressure-driven membrane operations, membrane distillation, osmotic distillation, integrated membrane processes

## Abstract

Pomegranate (*Punica granatum* L.) juice is well recognized for its high content of phytochemicals with proven health-promoting effects. Conventional processing techniques including clarification with fining agents, pasteurization and thermal concentration have significant influences on bioactive compounds and antioxidant activity of the juice. The growing consumers demand for high-quality pomegranate juice as well as the industrial interest for the production of functional foods, nutraceuticals, and cosmetics from its bioactive compounds have promoted the interest for minimal processing technologies. In this context, membrane-based operations represent an innovative approach to improve the overall quality of pomegranate juice production. This review focuses on the recent advances and developments related to the application of membrane technology in pomegranate juice processing. Conventional pressure-driven membrane operations and innovative membrane operations, such as osmotic distillation and pervaporation, are discussed in relation to their potential in juice clarification, fractionation, concentration and aroma recovery. Their implementation in integrated systems offer new opportunities to improve the healthiness and quality of the juice as well as to recover, purify and concentrate bioactive compounds for the formulation of functional ingredients.

## 1. Introduction

Pomegranate (*Punica granatum* L.), also called “seeded apple” or “granular apple,” is an ancient fruit-bearing deciduous shrub that belongs to the *Punicaceae* family. Due to its good adaptation to a wide range of climate and soil conditions, pomegranate is grown in many different geographical regions including tropical and subtropical regions [1]. Currently, Mediterranean countries, India, Iran and California are the main producers. India, China, Iran and Turkey have the largest area of production [2].

The genetic diversity of pomegranate is demonstrated by an excess of 500 globally distributed varieties, approximately 50 of which are known to be commercially cultivated [3].

The global pomegranate market was valued at USD 8.2 billion in 2018 and is expected to reach USD 23.14 Billion by year 2026, at a Compound Annual Growth Rate (CAGR) of 14.0 percent [4]. Increasing demand for pomegranate and its derivatives (such as pomegranate powder, pomegranate juice, functional beverages) as well as other pomegranate-derived products is driven by its widespread popularity as a functional food and a source of nutraceuticals [5,6].

Pomegranate juice is the major food product obtained from arils, which constitute about 50% of the fruit weight and contain about 78% juice and 22% seeds [7].

Pomegranate-based “superfruits” have become mainstream within the juice and functional beverage category thanks to their properties to deliver nutritional benefit and antioxidants into the diet [8].

**Figure 1:**
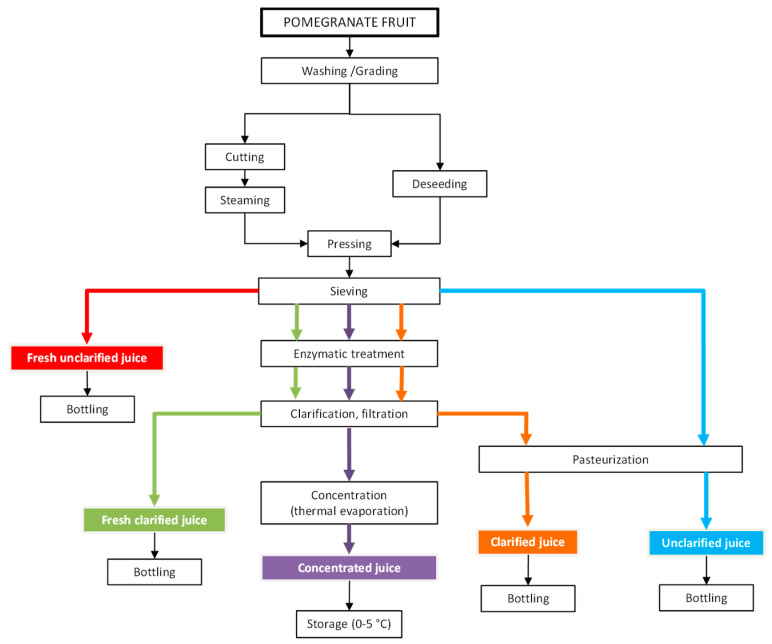
Flow diagram of processing operations for pomegranate juice production (adapted from [9]).

The production process of pomegranate juice, as reported in Figure 1, includes several steps such as washing, crushing, deshelling, pressing, clarification and pasteurization [9]. Traditional methods of clarification involve many steps, such as enzymatic treatment (depectinization), cooling, flocculation (gelatin, silica sol, bentonite and diatomaceous), decantation and filtration, which are labour and time consuming. In addition, these methods use large amounts of coadiuvants and additives with further drawbacks, such as the risk of dust inhalation caused by handling and disposal, environmental problems and significant disposal costs. Although the conventional thermal treatments do inactivate native enzymes and reduce the microbial load, they negatively impact the juice organoleptic properties. Flavour notes can be deteriorated, and juice darkening notes due to enzymatic browning and Millard reactions can occur. Off-flavour formation, colour change, reduction of nutritional values and high energy consumption are also typical drawbacks of thermal effects during evaporation processes for juice concentration [10]. Therefore, the use of mild technologies able to minimize the degradation of the functional molecules of the juice is strongly recommended to promote the development of high-quality products [11,12].

In this context, membrane separation processes represent a valid alternative to conventional juice clarification and concentration processes allowing efficient separation and concentration of solutes without changing phase and preserving chemical, physical and nutritional properties of food components. In particular, pressure-driven membrane operations, such as microfiltration (MF), ultrafiltration (UF), nanofiltration (NF) and reverse osmosis (RO), are today well consolidated operations in several agri-food applications, including fruit juice processing industry [13,14].

New membrane processes including membrane distillation (MD), osmotic distillation (OD) and pervaporation (PV) have been investigated in the last years for fruit juice concentration and recovery of aroma compounds [15].

The scope of the current review is to provide an outlook on the use of membrane technology in pomegranate juice processing in relation to the growing interest of researchers and manufacturers towards the preservation of the product’s physicochemical characteristics and shelf life. In particular, the use of conventional and innovative membrane processes in juice clarification, concentration and fractionation, as well as in the recovery of aroma compounds, is analysed and discussed in relation to their potential for the production of high quality and innovative products.

## 2. Juice Composition and Health Properties

Pomegranates produce a reddish-purple, moderately acidic juice containing in average 85.4% water and 15.6% dry substance, composed of sugars, organic acids, pectins, anthocyanins, polyphenols, vitamins and minerals. However, the juice composition, as well as organoleptic attributes, is strictly correlated to the pomegranate variety and juice production technology. Juices obtained from sarcotestas alone contain approximately 15% solids, lower than juices produced from whole fruits (16–17%). This is because sarcotestas are mainly sources of sugars, acids and minerals, while the rind is rich in polyphenolic compounds. On the other hand, juice pressed from whole fruits has a typical bitter taste due to the high content of phenolics [16].

The typical composition of three varieties of pomegranate juice (Wonderful, Chaca and Codpa) from squeezed arils of fresh fruits at the full ripe of maturity is reported in Table 1.

The antioxidant activity of the juice is much higher than that of red wine and green tea as well as of other juices including black cherry, cranberry, apple, orange and blueberry juices [18,19]. Thanks to this ability the juice is used as a natural bio-preservative as alternative to the use of synthetic antioxidants that have toxic side effects.

Pomegranate juice exhibits high antioxidant capacity because of its rich content of polyphenols, which represent the highest proportion of phytochemicals in pomegranate. In particular, the antioxidant activity of the juice can be attributed mainly to ellagic acid and its derivatives, anthocyanins and hydrolysable tannins [20].

The soluble polyphenol content in pomegranate juice varies within the range of 0.2–1.0%, depending on variety, including mainly hydrolysable tannins, ellagic acid derivatives and flavonoids.

Punicalagin, a large polyphenol with a molecular weight greater than 1000 belonging in the family of ellagitannins is responsible for more than the half pomegranate juice’s antioxidant effect [21].

Pomegranate juice is an important source of flavonoids including flavonols (catechin, epicatechin, gallocatechin) and anthocyanins. Anthocyanins are water-soluble plant pigments responsible of the red colour of both fruits and juice. They include 3-glucosides and 3,5-glucosides of delphinidin, cyanidin, and pelargonidin [22].

Phenolic acids include hydroxybenzoic acids (mainly gallic acid and ellagic acid) and hydroxycinnamic acids, principally caffeic acid, chlorogenic acid and p-coumaric acid [23,24]. Other chemical constituents in pomegranate juice include sugars (glucose, fructose, sucrose), organic acids (citric acid, malic acid, tartaric acid, fumaric acid, succinic acid, ascorbic acid, etc.), amino acids (proline, valine, methionine, glutamic acid, aspartic acid), indoleamines (tryptamine, serotonin, melatonin), tocopherols and minerals (Fe, Ca, Cl, Cu, K, Mg, Mn, Na, Sn and Zn) [25].

Pomegranate juice, being rich in bioactive compounds like polyphenols, has shown many health-related properties, such as antioxidant, anti-inflammatory, antihypertensive and antiatherogenic effects through in vivo and in vitro studies [26,27]. The healthiest constituents are ellagitannins, ellagic acid, punicic acid, anthocyanidins, anthocyanins, flavonols and flavones.

Ellagitannins have been associated to the prebiotic potential and antimicrobial activity of the juice [28]. In vitro studies have established the potential of pomegranate extracts as an antitumorigenic agent against various cancers including prostate cancer, renal cell carcinoma, papillary thyroid carcinoma cells and cervical cancer cell lines [29]. Mechanistic studies have revealed that Punica extracts and its components, individually or in combination, can modulate and target key proteins and genes involved in breast cancer [30]. A wide variety of molecules can be targeted by pomegranate to suppress tumour growth and metastasis, including those involved in cell-cell and cell-extracellular matrix adhesions, modulators of cytoskeleton dynamics and regulators of cancer cell anoikis and chemotaxis, pro-inflammatory and pro-angiogenic molecules [31].

Ellagic acid and punicalagin, have shown the ability to inhibit amine oxidases, α-glucosidase, dipeptidyl peptidase-4, lipase, triglyceride accumulation and adipogenesis-related genes, as well as to decrease lipogenesis and lipolysis in mouse and human adipose cells. These results support the development of functional foods for the prevention obesity, diabetes and dyslipidemias [32]. Studies with mice models have demonstrated that pomegranate juice exhibits a protective effect against oxidative damage in Parkinson’s disease as well as antileishmanial activity [33,34].

Finally, human studies have shown the beneficial effects of the juice on blood pressure, serum triglycerides, high-density lipoprotein (HDL) cholesterol, oxidative stress and inflammation in haemodialysis patients [35]. The juice consumption allows also to reduce the serum erythropoietin level [36] as well as systolic and diastolic blood pressure in patients with type 2 diabetes [37].

All these studies confirm the interest for the implementation of innovative technologies that provide minimum influence of juice processing and preservation on functional properties. These technologies represent also a useful approach for recovering phytochemical compounds from second-quality and over-ripe pomegranate fruits not suitable for commercialization, which can be exploited for the formulation of functional ingredients. The processing of these fruits wasted as by-products generates more profits and minimizes production losses along with the subsequent environmental benefits.

## 3. Fundamentals of Membrane Processes

### 3.1. Pressure-Driven Membrane Operations

Membrane processes are separation technologies based on the use of permselective membranes with the ability of transporting some components more readily than others. In pressure-driven membrane processes, the mass transport through the membrane is induced by a hydrostatic pressure gradient through the two sides (feed and permeate) of the membrane. As a result, the feed solution is converted in a *permeate* stream containing all components that have permeated the membrane and a *retentate* containing all compounds rejected by the membrane.

Pressure-driven membrane processes are commonly divided into four categories of increasing selectivity: microfiltration (MF), ultrafiltration (UF), nanofiltration (NF), and reverse osmosis (RO). MF is based on the use of symmetric or asymmetric membranes having pore size between 0.05 and 10 μm. It is used primarily to separate particles and bacteria from other smaller solutes. Operating pressures are of the order of 0.5–2 bar.

The UF process is based on the use of asymmetric membranes with pore size in the skin layer between 2 nm and 0.05 μm and operating pressures between 1 and 10 bar. It is used to separate viruses, colloids like proteins from small molecules like sugars and salts. Typically, UF membranes are characterized by the molecular weight cut-off (MWCO), defined as the equivalent molecular weight of the smallest species that exhibit 90% rejection. The MWCO of UF membranes is between 10^3^ and 10^6^ Dalton [38].

NF is characterized by a membrane pore size between 0.5 and 2 nm and operating pressures between 5 and 40 bar. It is essentially used to separate sugars, organic molecules and multivalent salts from monovalent salts and water.

The separation mechanism in MF, UF and NF processes is mainly the size exclusion. Other separation mechanisms include the electrostatic interactions between solutes and membranes, which depend on the surface and physiochemical properties of solutes and membranes.

RO membranes contain extremely small pores (in the range 0.1–1 nm) and are generally used to separate low molecular weight compounds from a relatively pure solvent. Operating pressures to obtain significant transmembrane flux can vary from 10 to 100 bar depending upon the osmotic pressure of the feed mixture. Therefore, RO has high-pressure requirements and consequently lower fluxes compared with other filtration processes. A solution-diffusion mechanism is mainly involved in the separation of compounds having different diffusivities and solubility in the membrane matrix [39].

The performance of a pressure-driven separation process is determined by the filtration rate and membrane separation properties. The separation capability of a membrane with respect to a component can be expressed in terms of membrane rejection as follows:

(1) $$R=\left(1-\frac{C_{i}^{p}}{C_{i}^{f}}\right)$$

where *R* is the rejection of the membrane for a specific component *i*, *C* is the concentration of component *i*, and superscripts *f* and *p* refer to the feed and permeate solutions, respectively.

Membranes are manufactured in a wide variety of materials. They include cellulose-based materials, synthetic polymers such as polyamide (PA), polyimide (PI), polysulfone (PS), polyethersulfone (PES), polyarylsulfone (PAS), polyethylene (PE), polyvinylidene difluoride (PVDF), polypropylene (PP), cellulose esters, polyvinylchloride (PVC), polytetrafluoroethylene (PTFE), polyacrylonitrile (PAN), nylon, polycarbonate (PC), inorganic materials and carbon fibres. These membranes are arranged into devices, called membrane modules, developed in order to fulfil important requirements such as high surface/volume ratio, low flow resistance, relatively low role of the polarization layer during operation and low construction costs.

Membrane modules are available in five basic designs, hollow fibre, spiral wound, tubular, plate-and-frame and capillary. They are quite different in their design, mode of operation, production costs and energy requirement for pumping the feed solution through the module.

### 3.2. Osmotic Distillation

Osmotic distillation (OD) is an attractive process for the concentration of liquid foods and various non-food aqueous solutions since it operates at low pressure and temperature, with minimal mechanical or thermal damage of the solutes [40]. This process is based on the use of a microporous, non-wettable membrane separating two liquid phases that differ greatly in terms of solute concentration. The hydrophobic nature of the membrane prevents penetration of the pores by aqueous solutions, creating air gaps within the membrane. The difference in solute concentration and consequently in water activity between the two sides of the membrane induces a vapour pressure difference that in turn leads to a water vapour transfer across the pores from dilute solution to the concentrated one [41,42,43].

Generally, the vapour transport through the membrane pores takes place via one of three molecular diffusion, Knudsen diffusion and viscous diffusion mechanisms, depending on the pore size of the membrane and the characteristics of the diffusing component [44].

Membranes used in OD are typically hydrophobic in nature. Typical polymers with low surface free energy such as PE, PTFE, PP and PVDF are used at this purpose.

Osmotic solutions used in OD should be thermally stable and also preferably non-toxic, non-corrosive and low cost. Inorganic salts such as MgSO_4_, NaCl, CaCl_2_, MgCl_2_, KH_2_PO_4_, K_2_HPO_4_ or organic solvents, including glycerol and polyglycerol, are typically used in OD applications.

### 3.3. Pervaporation

Pervaporation is a separation process in which a liquid mixture is separated by partial vaporization through a dense permselective membrane resulting in a vapour permeate and a liquid retentate. The driving force of PV is a gradient in chemical potential generated by the application of a partial vapour pressure difference between the liquid feed and the vaporous permeate. The difference in partial pressure is created on the permeate side, usually by vacuum generated by continuous pumping (vacuum pervaporation) or occasionally by sweeping with an inert gas (sweeping gas pervaporation). In both cases, the vapour permeate is converted into a liquid by means of a condenser [45].

PV membranes can be either hydrophilic, such as those realized in poly(vinyl alcohol) (PVA) and cellulose acetate, or hydrophobic made in polydimethylsiloxane (PDMS) or poly(trimethylsilypropyne) (PTMSP). Hydrophilic membranes favour the permeation of water whilst organic substances permeate preferentially through hydrophobic membranes.

According to the solution-diffusion model, the mass transfer of the permeants across the membrane occurs in three steps: (i) a selective absorption of solutes from the bulk feed liquid into the membrane at the feed side, (ii) a selective diffusion across the membrane owing the concentration gradient, and (iii) a desorption of vaporous permeate solutes on the permeate side.

Besides flux, two other parameters are often used to evaluate the performance of a pervaporation process. The separation factor, *α_i/j_*, describes the ability of the process to separate the substances *i* and *j*. It is expressed as:

(2) $$\propto_{i/j}=\frac{C_{i}^{p}}{C_{j}^{p}}\cdot \frac{C_{j}^{f}}{C_{i}^{f}}$$

where *C* represents concentration term, superscripts *p* and *f* represent feed and permeate, respectively, and *i* and *j* designate the two components of the liquid/liquid mixture to be separated

The corresponding enrichment factor (*β*) of a specific compound is defined as:

(3) $$\beta_{i}=\frac{C_{i}^{p}}{C_{i}^{f}}$$

These parameters are strongly depending on the process and its operating conditions (e.g., feed concentration, pressure, temperature, among others) [46].

## 4. Juice Clarification

Pomegranate juice in its original state contains only trace amounts of pectin. Therefore, it could be filtered easily after pressing without clarification. However, a clarification step is needed to prevent the formation of cloudy appearance during storage. In addition, the clarification step allows to reduce the bitter taste of the juice due to the high tannin content [47]. These polyphenols contribute to haze formation through mechanisms involving prior polymerization or condensation leading to the formation of polymeric complexes settling at the bottom of containers during storage. Nutritionists, however, recommend preserving these compounds during fruit juice processing, because they exert a protective effect on human health [48].

Traditional methods of fruit juice clarification involve many steps, such as enzymatic treatment (depectinization), cooling, flocculation (gelatin, silica sol, bentonite and diatomaceous), decantation and filtration, which present several drawbacks in terms of handling and disposal, long operating times, low yields and significant costs.

Membrane processes such as MF and UF represent a valid alternative to the conventional clarification procedures resulting in savings in labour and capital costs, short processing time, increased juice yield and avoidance of fining agents and filter aids [49]. Additionally, MF and UF are very efficient in the preserving the juice freshness, aroma and nutritional value while obtaining high-quality, natural fresh-tasting and additive-free products as the separation process requires no heat application or the use of chemical agents [50].

Permeate fluxes and quality of the clarified juice are strongly affected by operating conditions, such as crossflow velocity (CFV); transmembrane pressure (TMP); temperature; volume reduction factor (VRF) and membrane properties such as MWCO/pore size, configuration and material. In particular, high permeate flux is necessary for filtration to be practical and economic, and product quality should at least meet those obtained by the other standard clarification methods.

Typical applications of MF and UF membranes in pomegranate juice clarification are summarized in Table 2.

### 4.1. Effect of Operating Conditions and Membrane Properties on Permeate Flux and Juice Quality

Mirsaeedghazi et al. [72] evaluated the physicochemical properties of pomegranate juice clarified with different flat sheet membranes (PVDF with pore size 0.22 and 0.45 μm; mixed cellulose esters with pore size of 0.22, 0.1 and 0.025 μm). The selected membranes removed all suspended solids of the raw juice and reduced more than 99% of the juice turbidity. The titratable acidity was reduced of about 20% without modifying the pH of the juice. This reduction was attributed to blockage of acidic components in the cake layer on the membrane surface. Total soluble solids were also removed with a mean rejection factor of 20.8%. Total anthocyanins and ellagic acid were reduced from 25% to 40% depending on the used membrane. The rejection towards total phenolics was in the range 30–50%.

Cassano et al. [66] investigated the effect of transmembrane pressure (TMP) and cross flow velocity (CFV) on permeate flux, membrane fouling and quality of pomegranate juice clarified with modified poly ether ether ketone (PEEK-WC) and PS hollow fibre membranes prepared in laboratory. For both membranes, the increase of CFV reduced concentration polarization phenomena increasing the permeate flux. On the other hand, an increase in pressure was not efficient in terms of flux improvement due to the thickening or compression of the deposited layer onto the membrane surface and adsorption in the membrane pores. A limiting flux was reached when the transmembrane pressure was increased up to a specific value; after that, any further increase determined no significant increase of the permeate flux. PS membranes exhibited lower indexing of fouling and lower rejection towards bioactive compounds in comparison to PEEK-WC membranes.

PVDF membranes in hollow fibre configuration with pore size of 0.13 μm presented higher permeation fluxes and lower retention towards bioactive compounds, including flavonoids and anthocyanins, in comparison to PS hollow fibre membranes with similar pore size [67]. Consequently, the PVDF clarified juice exhibited greater antioxidant effects than the juice clarified with PS membranes. In addition, the treatment with PVDF membranes enriched the juice in α-amylase and α-glucosidase inhibitors.

The different selectivity of PVDF and PS membranes towards bioactive compounds of pomegranate juice can be attributed to the chemical nature of both polymers. PS is made of a carbon chain alternating aromatic and aliphatic units, responsible for the hydrophobic character of the polymer, and oxygen and sulphur dioxide subunits providing the hydrophilic character of the polymer. On the other hand, PVDF is made of alternating units of CH_2_ and CF_2_, conferring a hydrophobic nature to the material. The hydrophilic subunits of PS could be, therefore, more prone to create hydrogen bonds and Van-der-Waals interactions with the hydroxyl groups exhibited by polyphenols, flavonoids and anthocyanins with consequent adsorption of these components at membrane surface and the formation of fouling layers. PVDF, on the contrary, is less susceptible to hydrogen bonds and Van-der-Waals interactions making it more resistant to fouling and highly permeable to the considered compounds.

The clarification of pomegranate juice with a flat-sheet PVDF membrane with pore size of 0.22 μm was modelled computational fluid dynamics (CFD) in order to predict velocity and pressure patterns in the membrane module during the process [55]. Results indicated that the fluid velocity was zero near the upper wall and increased toward the centre of channel in the early stage of the process. In addition, the feed pressure was increased as soon as the feed entered the membrane module. According to these results, membrane damages can be minimized if the feed flow is parallel to the membrane surface at the entrance of the module in order to reduce the pressure created in vertical patterns.

The use of computational fluid dynamics was also investigated to simulate the effect of the feed canal height on permeate flux [64]. Results of simulation showed that the volumetric flow rate of permeate increased by increasing the feed canal height. Experimental results obtained in the clarification of the juice with membrane units having different canal heights (0.4 and 2 cm) were in agreement with the simulation results.

Recently, Beaulieu et al. [73] evaluated the quality of “Wonderful” pomegranate juice produced by hydraulic pressing, UF and high temperature short-time pasteurization stored at 4 and 25 °C for 3 months. All these processes had minor effects on colour parameters, organic acids and anthocyanidins. The UF process functioned well and was rapid compared to older complicated fining and filtration methods used to reduce sedimentation and clarify juice while removing polymeric compounds.

According to Colantuono et al. [70], the use of 0.45 µm mixed cellulose ester MF membranes is able to reduce all lactic acid bacteria and yeasts/moulds of the raw juice below the detectable threshold (<1 CFU mL^−1^). Therefore, MF is comparable to pasteurization in guarantee the microbiological stability of the juice, avoiding spoilage of the final product.

In addition, in the clarified juice, the monitored microbial groups remained below the detectable threshold over 28 days of storage. MF reduced the turbidity of the final product about 280-fold compared with the raw juice improving both clarity and colour of the juice. The clarified juice showed the highest level of monomeric anthocyanins and the lowest levels of copigmented and polymeric anthocyanins in comparison with raw and pasteurized juices, probably because copigmented anthocyanins were partially removed during MF.

Recently, Morittu et al. [71] evaluated the in vitro antioxidant and hypoglycemic activities of raw pomegranate juice and clarified juice with PVDF hollow fibre membranes. The clarified juice resulted more active than untreated juice in all antioxidant tests (DPPH, FRAP and -carotene blenching tests). Therefore, the clarification process seems to produce a positive effect on the antioxidant activity despite a reduction of phenols content of pomegranate juice. This suggests that the filtration process retains some compounds that may limit the absorption of pomegranate juice, reducing the juice beneficial properties [74]. The untreated juice was more active than the clarified juice in inhibiting α-amylase, while α-glucosidase was mainly inhibited by the clarified juice.

### 4.2. Juice Pre-Treatment

Juice pre-treatment is an appropriate method to decrease the content of macromolecules that are able to accumulate on the membrane surface or in the membrane pores so reducing membrane fouling in the membrane processing of fruit juices. In particular, pectic substances are identified as the major hindrance to filtration performance, because they form a compressible gel layer that could lead to severe fouling of MF and UF membranes.

Pre-treatment methods to increase the performance of membrane clarification include the use of pectolic enzymes, filtering aids (i.e., gelatin, bentonite) and centrifugation [75,76,77].

Yousefnezhad et al. [69] found that centrifugation at 2000 rpm for 5 min increased the permeate flux in the MF of pomegranate juice with mixed cellulose ester membrane with pore size of 0.22 μm; increasing the centrifugation time up to 10 min extensively increased the permeate flux. On the other hand, centrifugation at 4000 rpm showed a negative effect on the permeate flux. In both cases, centrifugation did not change nutritional value of pomegranate juice except polyphenol content.

The effect of different pre-treatment processes with fining agents, including gelatin, bentonite and polyvinyl polypyrrolidone (PVPP), on the clarification of pomegranate juice with a PVDF UF membrane of 30 kDa was investigated by Bagci [62]. The whole results indicated that a sequential application of PVPP and bentonite produced the best performance in terms of permeation flux and recovery of the hydraulic permeability of the UF membrane after cleaning. According to the resistance data, this approach allowed to reduce at a greater extent the formation of cake layer in comparison with other pre-treatment systems. The use of PVPP produced also a greater adsorption of low molecular weight phenolics, such as monomeric anthocyanins and catechins, improving the juice clarity and preventing oxidation and polymerization reactions leading to turbidity, browning and astringent flavour formation during further storage. Higher anthocyanin adsorption capacity of PVPP in comparison with gelatin has been also reported for conventional clarification of pomegranate juice [47].

Neifar et al. [57] evaluated the effect of the enzymatic pre-treatment with laccase on the total phenol content and clarity of pomegranate juice ultrafiltered with a tubular ceramic membrane (Carbosep M2, Orelis, Miribel, France) with a MWCO of 15 kDa. A central composite design allowed to find the optimal conditions (enzyme concentration 5 U mL^−1^; incubation time 300 min; incubation temperature 20 °C) to produce a clear and stable juice. The optimized conditions led to a total phenol reduction by about 40% accompanied by a decrease in the juice clarity (about threefold) and an increase in the juice colour (about twofold) in comparison with the untreated juice (polyphenols concentration, 1681 mg L^−1^; clarity, 130; colour, 380).

### 4.3. Analyses of Membrane Fouling

Membrane fouling is one of the most important limitations against industrialized use of membrane clarification. This phenomenon is caused by the accumulation of macromolecular or colloidal species (such as pectins and proteins) on the membrane surface (concentration polarization and gel layer) or by physico-chemical interactions with the membrane such as adsorption on the membrane pore walls and pore plugging [78]. The degree of membrane fouling determines the frequency of cleaning, lifetime of the membrane and the membrane area needed; therefore, it has a significant effect on the cost, design and operation of membrane plants.

**Figure 2:**
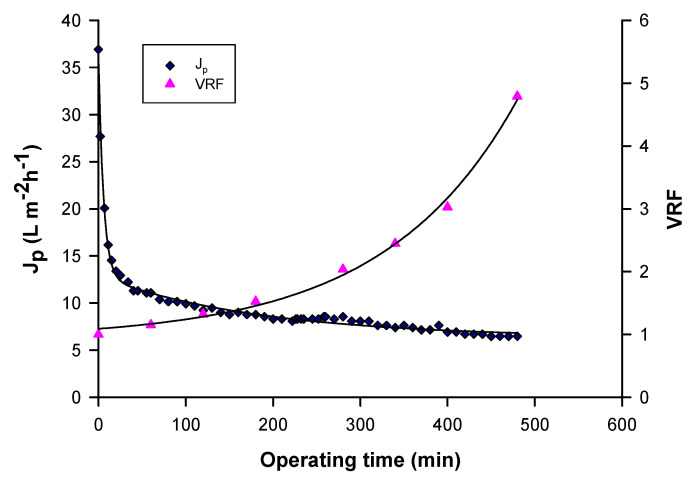
Time evolution of permeate flux and volume reduction factor (VRF) in the clarification of pomegranate juice with hollow fibre UF membranes (Operating conditions: TMP, 0.6 bar; Q_f_, 400 L/h; T, 25 ± 1 °C) [68].

Figure 2 shows the time evolution of permeate flux and VRF in the clarification of raw pomegranate juice with a cellulose triacetate UF membrane module of 150 kDa (FUC 1582, Microdyn Nadir, Wiesbaden, Germany) in hollow fibre configuration. Fouling phenomena led to a gradual decline of permeate flux from the initial value of about 37 L m^−2^ h^−1^ up to reach a steady-state value of 7 L m^−2^ h^−1^ [68].

Several mathematical models have been proposed to analyse the fouling mechanism in membrane filtration. Empirical mechanistic models are based on the modified form of the Hermia’s law developed for constant pressure dead-end filtration [79].

According to the Field model [80] the permeate flux decline with time is described by the following differential equation:

(4) $$\frac{dJ}{dt}=k\cdot \left(J-J_{lim}\right)\cdot J^{\left(2-n\right)}$$

in which *J_lim_* represents the limit value of the permeate flux obtained in steady-state conditions; *k* and *n* are phenomenological coefficient and general index, respectively, depending on fouling mechanism. In the complete pore blocking (*n* = 2), it is assumed that particles are larger than pore size, and a complete pore obstruction is obtained. The intermediate pore blocking (*n* = 1) is a dynamic situation of blocking/unblocking in which particles may bridge a pore by obstructing the entrance but not completely blocking it. In standard pore blocking (*n* = 1.5), particles are much smaller than the membrane pore diameter and can enter the pores reducing the pore volume. The cake filtration (*n* = 0) occurs when particles are larger than the membrane pore diameter, and a cake layer is formed on the membrane surface. In this case the overall resistance to the mass transport is composed of the cake resistance and the membrane resistance, which is assumed to remain unchanged.

The resistance-in-series model is another approach commonly used to analyse the flux decline in the filtration of real feeds. According to this model, both concentration polarization and membrane fouling occur to add additional resistances to the membrane and hence to the permeate to pass through [81]. The equation is expressed as follows:

(5) $$J_{p}=\frac{TMP}{\mu (R_{m}+R_{p}+R_{f})}$$

where *J_p_* is the permeate flux (m s^−1^), TMP is the transmembrane pressure (kPa), *μ* the fluid viscosity (Pa s), *R_m_* is the membrane resistance (m^−1^), *R_p_* the polarized layer resistance (m^−1^) and *R_f_* the fouling resistance (m^−1^).

Mirsaeedghazi et al. [51] found that the formation of a cake layer is the dominant fouling mechanism in the clarification of pomegranate juice with MF membranes in PVDF and mixed cellulose ester with pore size of 0.22 μm and 0.1 μm, respectively. The cake layer formation is a reversible phenomenon and can be removed by cleaning of the membrane with water at high velocity and low pressure. On the other hand, complete blocking or standard blocking are not reversible; therefore, membrane should be cleaned before they occur.

PVDF membranes with pore size of 0.45 μm showed higher permeation flux than that measured for membranes with pore size of 0.22 μm in similar operating conditions [52]; however, the degree of flux decline was greater for membranes with smaller pore size. The fouling index for both membranes decreased over time since cake formation, as a major reason of flux decline, was created in the first stages of juice filtration. During the process, cake formation is replaced with other mechanisms that have fewer effects on membrane fouling.

Similar results were found in the evaluation of membrane fouling of MF and UF membranes in mixed cellulose ester with pore size of 0.22 µm and 0.025 µm [58]. In particular, greater fouling resistances were observed for the UF membrane due to its smaller pore size. The fouling resistance of the UF membrane was reduced significantly after the treatment of the juice with the MF membrane.

A mathematical model was developed by Mirsaeedghazi et al. [53] to analyse changes in concentration in the cake polarization layer as a function of time and distance to the membrane surface in the clarification of pomegranate juice with a mixed cellulose esters flat membrane of 0.1 μm (Milipore, Billerica, MA, USA). The model showed that the solute concentration increased with the increase of operating time and TMP and the decrease of distance to the membrane surface and feed velocity. The predicted values matched the experimental data; therefore, the model can be used to predict the concentration profile in the cake layer for each condition.

Baklouti et al. [59] applied the Hermia model to describe the flux decline during the UF of untreated and laccase treated pomegranate juice with a tubular inorganic membrane module of 15 kDa (Carbosep M2, Orelis, Miribel, France). The fouling mechanism resulted affected by the pre-treatment process. In particular, cake formation was found as the main mechanism responsible for membrane fouling for untreated juice. On the other hand, the complete pore blocking resulted the predominant fouling mechanism for the enzymatically treated juice.

Total phenolic rejection decreased from 45% to 21% when the TMP raised from 1 to 2 bar due to the higher amount of solute flowing through the membrane. A further increasing in TMP from 2 to 3 bar resulted in an increased amount of deposited fouling materials on the membrane surface and consequently of the total phenolic rejection. The slow decline of permeate flux in batch concentration experiments at a TMP of 2 bar and CFV of 1 m s^−1^ was attributed in the early stage of UF to internal fouling. The pre-treatment improved the permeation flux, and steady-state flux values of about 37 L m^−2^ h^−1^ were reached in comparison to 25 L m^−2^ h^−1^ of the untreated juice.

The response surface methodology was also used to define optimal operating conditions for juice UF treatment [60]. Experiments were performed according to Box–Behnken design by changing the levels of TMP, feed flow rate and temperature. The studied responses were fouling resistance (Rf), concentration polarization resistance (Rcp) and permeate limit flux. According to the desirability function approach the selected UF conditions of the compromise were identified (TMP, 3 bar; feed flow rate, 0.95 L min^−1^; temperature, 30 °C). Optimal values of R_f_, R_cp_ and permeate limit flux were equal to 18%, 72% and 19 L m^−2^h^−1^, respectively.

Sharifanfar et al. [63] evaluated the effect of the feed canal height on the permeate flux and fouling mechanism in the clarification of pomegranate juice with a flat sheet MF membrane of 0.22 μm. Permeate flux increased by increasing the canal height from 0.4 to 2 cm and from 1.5 to 2 cm. Total and irreversible fouling resistances also increased by increasing the canal height; this was attributed to the greater volume of feed over the membrane surface and a greater Re when the canal height is increased.

Cake formation resulted the dominant mechanism of fouling for each feed canal height. However, for canal height of 1.5 and 2 cm, cake formation occurred at the beginning of the filtration while for a canal height of 0.4 cm, cake formation occurred at the end of the process.

A mathematical model to quantify the flux decline behaviour during MF of pomegranate juice with hollow fibre membranes under turbulent flow conditions was developed by Mondal et al. [61]. Theoretical results were compared with experiments data of juice clarification with poly (ether ether ketone) and polysulfone hollow fibre membranes. Gel layer resistance had significant impact on permeate flux. Under the total recycle mode of filtration, the gel layer thickness decreased with turbulence resulting in an increase of permeate flux. A different behaviour was observed under a batch concentration operation. In this case, a transition time was identified after 3 h of operation, after which the gel layer thickness became significantly high and gel resistance became more dominant due to increase in feed concentration compared to turbulence in flow channel.

The model successfully predicted the performance of batch mode of filtration, and the agreement with experimental data was within ±5%.

Several attempts have been performed to limit membrane fouling mechanisms and increase the performance of membrane filtration. They include the use of magnetic particles, electric fields, gas sparging techniques, rotation and vibration systems and ultrasound waves [82,83,84,85] in order to inhibit the interaction of foulant particles with membrane.

Aghdam et al. [65] evaluated the effect of ultrasonic treatment on different fouling mechanisms in the clarification of pomegranate juice with a 0.45 μm hydrophilic mixed cellulose ester membrane. Cake formation resulted the dominant mechanism in both processes with and without ultrasonic treatment; however, the cake layer thickness in the membrane process in ultrasonic bath was much lower than in absence of ultrasonic waves (1.5 μm against 6 μm). In particular, in the treatment with ultrasounds cake formation was the main fouling mechanism in the early stages of filtration and changed to other fouling mechanisms such as intermediate blocking in the last stages of the process.

In another study, Aghdam et al. [86] found that permeate flux increased with ultrasonic treatment. However, this phenomenon was partially attributed to the increased feed temperature, rather than ultrasound waves. Analyses of membrane fouling mechanism revealed that ultrasound waves reduced total resistance through the reduction of both irreversible fouling and cake resistances. Despite a positive effect on membrane fouling, the ultrasonic treatment produced a significant reduction of the anthocyanin content and of the antioxidant activity due to the increased temperature of the juice. Therefore, cold membrane treatment in an ultrasonic bath was proposed to preserve the juice quality.

## 5. Juice Fractionation

The recovery and purification of bioactive compounds, particularly soluble polyphenols from food processing streams and by-products, have attracted considerable economic interest in the last years. Pressure-driven membrane operations also combined in a sequential form or with other separation technologies offer new opportunities and perspectives for recovering efficiently phenolic compounds without losing their activity [87]. In particular, tight UF membranes (1–3 kDa) and NF membranes have been widely explored in the last years for the recovery of phenolic compounds from several agri-food by-products and vegetable extracts [88]. These membranes display high separation performance in concentrating low molecular weight phenolic compounds and separation of phenolic compounds from sugars [89].

In the light of these properties, Conidi et al. [68] investigated the use of flat-sheet UF and NF membranes with MWCO ranging from 1 to 4 kDa for the recovery of phenolic compounds from clarified pomegranate juice. Among the selected membranes the Desal GK membrane (from GE Osmonics, Minnetonka, MN, USA), with a MWCO of 2000 Da, displayed higher permeate fluxes, lower fouling index and a good separation efficiency of sugars from phenolic compounds in comparison with the other tested membranes. For this membrane, a linear increase of the permeate flux, from 7 to 40 kg m^−2^ h^−1^, was observed by increasing the TMP from 5 to 25 bar. The absence of a limiting flux was attributed to the preliminary treatment of the raw juice by UF, which removed most part of suspended solids. The retention of phenolic compounds was higher than 90%, independently of the operating pressure.

**Figure 3:**
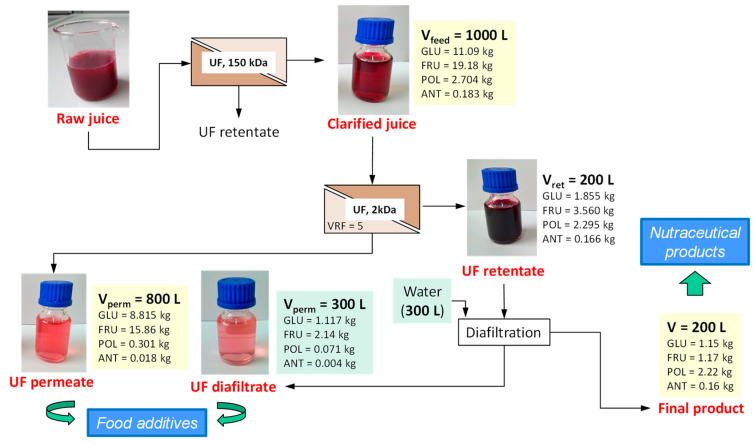
Mass balance of the fractionation process of the pomegranate clarified juice with Desal GK membrane (GLU, glucose; FRU, fructose; POL, polyphenols; ANT, anthocyanins) [68].

A retentate fraction enriched in phenolic compounds was obtained through concentration experiments up to volume reduction factor (VRF) of 5 followed by constant volume diafiltration experiments performed in a discontinuous way in order to improve the removal of glucose and fructose from the UF retentate. The proposed process, depicted in Figure 3, produced yields of polyphenols and anthocyanins in the retentate stream of about 84.8% and 90.7%, respectively. The diafiltration step allowed to reach a recovery efficiency in the permeate side for glucose and fructose up to 90% and 93%, respectively.

## 6. Juice Concentration

### 6.1. Nanofiltration and Reverse Osmosis

The concentration of fruit juices is a key unit operation in fruit processing aimed at removing water to extend the shelf life significantly and to ensure easier transportation. Thermal evaporation is the most widely used technique to remove water from fruit juices. This process is proved to induce adverse effects on sensorial, nutritional and chemical properties of final products particularly because of its destructive thermal effect. In particular, the production of pomegranate juice concentrate by evaporation processes such as microwave heating, rotary vacuum evaporator and heating at atmospheric pressure produces a significant reduction of the colour parameters (lightness (L), red/green coordinate (a) and yellow/blue coordinate (b)) and the appearance of a typical reddish brown colour [10]. In addition, the degradation rate of pomegranate anthocyanins and antioxidant capacity increases with pressure and temperature in both microwave and conventional heating methods [90].

Cryoconcentration, in which water is removed as ice rather than as vapor, is an alternative technique to thermal evaporation with high potential for preserving nutritional and sensory properties of fruit juices. The use of complete block cryoconcentration process was efficient in preserving the attractive red colour of pomegranate juice in comparison to thermal processes [91]. Results indicated that the colour values (L, a, b, ΔE) of concentrated and ice fractions were affected by cryoconcentration stage while freezing temperature and thawing mode did not have a significant effect on these factors.

Despite significant advantages over conventional thermal evaporation, freeze concentration systems are limited by high energy consumptions. Besides, the achievable concentration (about 50 °Brix) is lower than the values obtained in thermal evaporation (60–65 °Brix).

Membrane processes such as NF, RO and OD allow to concentrate juices at mild low-temperature conditions, reducing energy consumption and preserving aroma, nutritional and bioactive compounds [92].

RO membranes are characterized by high selectivity and solute retention. However, the osmotic pressure and viscosity of fruit juices increase rapidly with the increasing of the sugar concentration affecting remarkably the productivity of the process. In addition, the quality of the juice can be damaged at high operating pressures. Therefore, considering that the final concentration that can be reached by RO is limited (generally up to 30 °Brix), this technique can be useful as a pre-concentration step before a final concentration with other technologies [93].

Recently, Bagci et al. [94] investigated the use of polyamide (PA) thin film composite (TFC) RO membranes in flat-sheet configuration (UTC 73 U from Toray Membrane, Poway, CA, USA) for the preconcentration of clarified pomegranate juice before further concentration by OD. During RO, total soluble solids (TSS) increased up to 18 °Brix. The increase in osmotic pressure (approximately of 27 bar), relatively close to the hydraulic pressure (30 bar), did not allow to reach higher concentration. In order to improve their anti-fouling characteristics, PA membranes were submitted to a low-pressure nitrogen plasma (LPNP) activation and their performance was compared with that of plain membranes. Experimental results indicated that permeate fluxes of plasma modified membranes were much higher than those achieved with plain membranes (11.31 kg m^−2^ h^−1^ against 3 kg m^−2^ h^−1^ at the end of the process). In addition, the TSS content achieved using modified membrane was comparatively higher than that achieved using the plain membrane at any time during the process. The higher permeation flux of LPNP modified membranes was attributed to their increased hydrophilicity promoting the sorption of water on the membrane surface [95].

The NF process offers specific advantages over RO in fruit juice concentration mainly due to the lower operating pressures of the process, which allows to reduce the energy consumption (21% lower than RO) and improve the juice quality [96].

The concentration of pomegranate juice by NF was investigated by Mirsaeedghazi et al. [97] by using a polyethersulfone spiral-wound membrane with a MWCO of 5kDa (Permionics, Ltd., Vadodara, India). The juice previously clarified by UF was concentrated up to 19 °Brix. Analysis of membrane fouling revealed that *R_c_*, and *R_frev_* contribute greatly to the *R_t_* (36.1% and 44.1%, respectively), and these can be removed by washing methods. Critical flux (the flux where the transition between concentration polarization and fouling occurs) and limiting flux (where irreversible fouling occurs locally on the membrane surface) values were of 6.0 × 10^−6^ m s^−1^ and 9.069 × 10^−6^ m s^−1^, respectively.

### 6.2. Osmotic Distillation

OD is an attractive process for the concentration of solutions containing thermo-sensitive compounds such as fruit juices and pharmaceuticals since it allows to reach high levels of total soluble solids (up to 65–70 °Brix) at low operating temperature and pressure, thereby maintaining original organoleptic and sensorial characteristics of the raw material [92,98].

The use of OD has been also investigated in pomegranate juice concentration, and most applications refer to the use of clarified juice. It is well known that the clarification of the raw juice allows to improve the OD flux due to the reduction in the viscosity of the concentrated juice-membrane boundary layer where the solute concentration is the highest [99].

Evaporation fluxes in OD are affected by several parameters including the solute content, the temperature and the flowrate of both juice and stripping solution as well as the membrane properties (i.e., membrane thickness, porosity, etc.).

Cassano et al. [56] investigated the potential of OD in the concentration of ultrafiltered pomegranate juice (with a TSS content of 16.2 °Brix) up to 52 °Brix. The clarified juice was recirculated in the shell side of a polypropylene hollow fibre membrane module (Liqui-Cell Extra-Flow 2.5 × 8-in. membrane contactor, Membrana, Charlotte, NC, USA) with an average pore diameter of 0.2 μm and a total membrane surface area of 1.4 m^2^; calcium chloride dihydrate solution 10.2 M, used as stripping solution, was pumped through the lumen side.

The process was operated at a TMP of 0.4 bar and a temperature of 25 ± 2 °C. Evaporation fluxes decreased in the first part of the process from 0.85 to 0.55 kg m^−2^ h^−1^ due to the dilution of the brine solution, and consequently, to the decrease of driving force for water transport through the membrane. The further decrease of evaporation fluxes up to 0.38 kg m^−2^ h^−1^ was mainly attributed to the increased concentration of the juice. Therefore, at concentration values higher than 30 °Brix evaporation fluxes were mainly affected by the juice viscosity.

**Figure 4:**
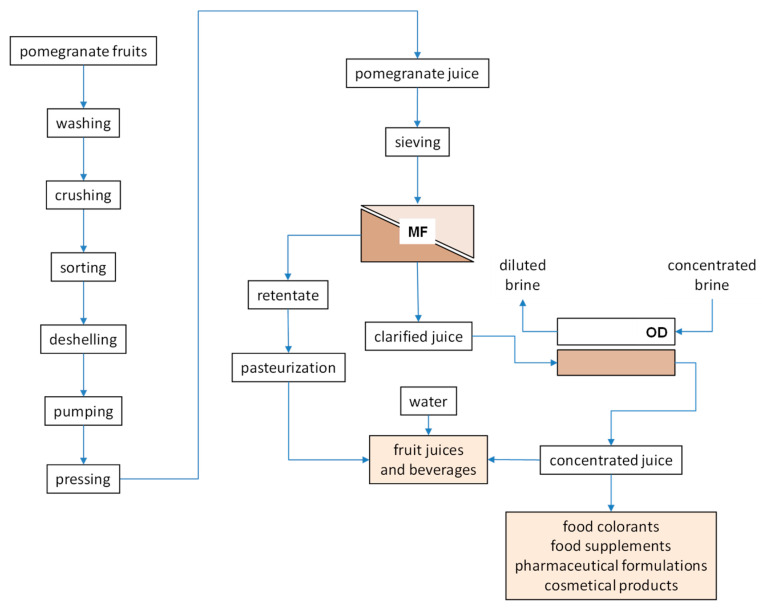
Integrated membrane process for the production of high-quality concentrated pomegranate juice (adapted from [56]).

Similar results have been reported in the concentration of kiwifruit [100], melon [101], cactus pear [102] and passion fruit [103] juice by OD. The antioxidant activity of the juice, attributed to a great extent to total phenols and anthocyanins content, was efficiently preserved during the concentration step independently by the TSS content reached in the process. An integrated membrane process, schematically depicted in Figure 4, was proposed on the basis of the results obtained on laboratory scale.

**Figure 5:**
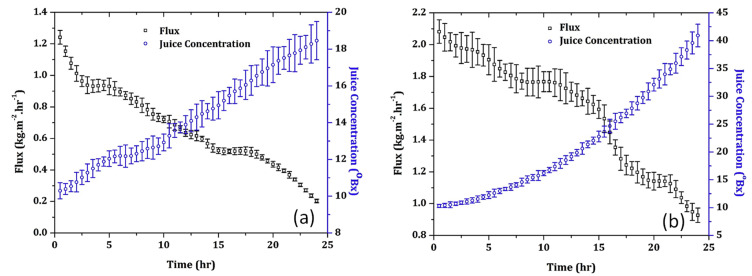
Concentration of clarified pomegranate juice with flat-sheet (**a**) PVDF and (**b**) PTFE membranes (Temperature, 25 ± 0.50 °C, feed and stripping solutions flow rate, 0.012 m^3^ h^−1^, stripper concentration: 6 M) [105].

A Liqui Cel^®^ 1.7 × 5.5 in. PP hollow fibre mini module (Membrana, Charlotte, NC, USA) was also used by Rehman et al. [104] to concentrate pomegranate juice by using CaCl_2_ 6M as stripping solution. The juice, previously clarified with PP spun filter (pore size 5 µm), was concentrated up to 52.0 °Brix, reaching a maximum flux of 1.053 kg m^−2^ h^−1^ at a temperature of 25 °C. Feed and stripper flow were in laminar range, so the concentration polarization layer had a constant thickness creating an additional resistance to mass transfer which resulted in a flux decline during the OD process. For spent membranes after 48 h of operation, besides the concentration polarization layer, the deposition of residual solids on the membrane surface blocked the pores leading to a higher flux decline. SEM analyses of fresh and spent membrane fibres after 48 h of operation revealed a thick and continuous fouling layer on the membrane surface due to a combination of suspended particles and the organic layer that remained unaffected after the cleaning process. Several compounds of the juice, including sugars, anthocyanins, polyphenols and organic acids, were considered as organic foulants. In another study Rehman et al. [105] compared the performance of flat-sheet PVDF and PTFE membranes with pore size of 0.2 μm (TS Filter Membranes, China) in the OD concentration of clarified juice by using CaCl_2_·2H_2_O 6M as stripping solution. The PTFE membrane showed higher evaporation fluxes allowed to reach higher juice concentration (41 °Brix) in a processing time of 24 h in comparison to the PVDF membrane (18 °Brix) (Figure 5). The hydrophobicity of the PVDF membrane declined by 29% after juice concentration, as opposed to 6% with the PTFE membrane, indicating that this membrane is much prone to wetting phenomena. Coating of hydrophobic PVDF hollow fibres with chitosan has been shown to protect membranes against wetting and flavour loss in the OD process maintaining stable flux [106].

Evaporation fluxes in OD can be significantly improved through a combination of chemical and thermal gradients through a hydrophobic membrane: the resulting process is defined as osmotic membrane distillation (OMD) [107].

Onsekizoglu [108] evaluated the performance of both OD and OMD processes in the concentration of clarified pomegranate juice by using a capillary polypropylene membrane module (MD 020 CP 2N, Microdyn-Nadir, Wiesbaden, Germany). The clarified juice was recirculated in the shell side of the module, while calcium chloride dihydrate at 65% (w/w) was recirculated in the tube side in a counter-current mode. In the OMD process, a temperature difference was imposed between the stripping solution (10 ± 1 °C) and the juice (30 ± 1 °C) in order to provide an additional driving force.

Evaporation fluxes in OMD resulted higher that those measured in OD allowing higher concentrations to be reached in shorter periods of juice processing. In particular, the clarified juice with a TSS content of 17.1 °Brix was concentrated in OD up to 55.5 °Brix with an average evaporation flux of about 0.65 kg m^−2^ h^−1^; in OMD a concentrated juice at 57.4 °Brix was produced with an average evaporation flux of 2.2 kg m^−2^ h^−1^.

The impact of both processes on product quality was also investigated and compared with that of thermal evaporation (TE). The physico-chemical characteristics of untreated, clarified juice and the concentrated juices produced by OD, MOD and TE are reported in Table 3. The overall results indicated that OD and OMD resulted very efficient in preserving the original characteristics of the juice including colour, total antioxidant activity (TAA), phenolic compounds and organic acids. On the other hand, TE produced a considerable loss in natural colour and TAA and a marked formation of hydroxymethyl furfural (HMF).

The application of the *Friday 13*th framework to a two-step integrated UF-OD process demonstrated with independent data for pomegranate juice showed that membrane fouling occurred in 10.5% of all operations [109]. Experimental results indicated that a reduction in vulnerability of the global process combined with an improvement of process reliability could be achieved through a depectinization of the fresh juice, before the UF step as well as through the introduction of improved process control to reduce the naturally occurring fluctuations in filtration time.

Recently, Roozitalab et al. [44] evaluated the influence of operating parameters on the performance of nanofibrous polyether-block-amid (PEBA) membranes in the OD concentration of raw juice by using calcium chloride dihydrate as osmotic solution. Membranes were prepared by the electrospinning technique of PEBA solutions on a non-woven polyester substrate. Their average diameter was 50–150 nm; mean pore diameter and porosity were 836 nm and 70.9%, respectively. Transmembrane fluxes increased by increasing the concentration of the osmotic solution: a salt concentration of 5.43 M produced an evaporation flux of 1.18 kg m^−2^ h^−1^ that was 31% higher than the flux at a salt concentration of 4.62 M. The combination of a chemical gradient with a thermal gradient between the juice and salt solution also enhanced the water vapor flux through the membrane. Considering that the membrane pore size was larger than the mean free path of the water vapor molecules (calculated as 0.3 nm), a molecular diffusion mechanism of water vapor molecules through the membrane pores was suggested. Therefore, the increased transmembrane flux at higher temperature differences between juice and osmotic solution was attributed to the increased water vapor diffusion coefficient through the membrane pores. However, considering the detrimental effect of the temperature on juice quality, the concentration of raw juice was performed in isothermal conditions.

**Figure 6:**
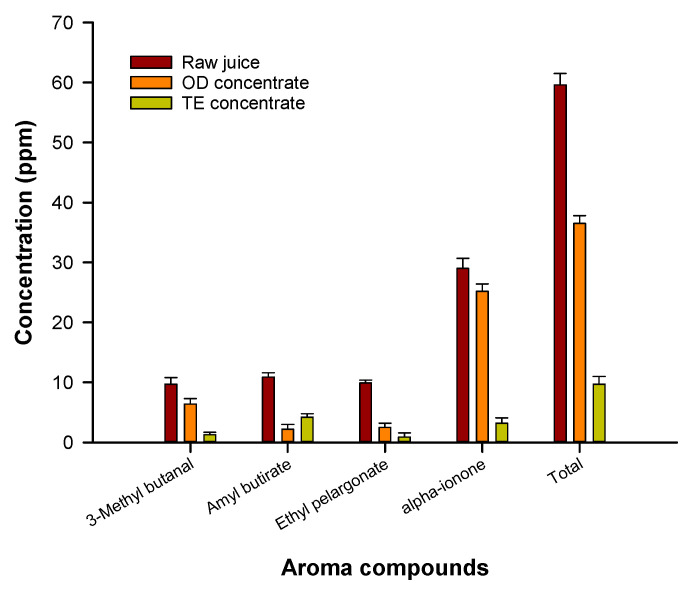
Aroma compounds in pomegranate juice and juice concentrated by osmotic distillation (OD) and thermal evaporation (adapted from [44]).

Electrospun nanofibrous PEBA membranes with a thickness of 60 μm produced higher evaporation fluxes in comparison to thinner membranes of 30 μm. Although the intrinsic membrane resistance against the mass transport increases with the membrane thickness, this result was attributed to the effect of temperature polarization on the vapor pressure difference across the membrane and on the permeation flux. Indeed, a decrease in the membrane thickness enhances the heat loss from the membrane hot side to the membrane cold side leading to a reduction of the driving force which results in a lower transmembrane water flux. The quality of the OD concentrated juice was compared with that of thermally evaporated juice at a temperature of 73 °C, in terms of retention of volatile aroma compounds and phenolic compounds. Figure 6 shows the analytical evaluation of aroma compounds identified in the raw juice and in both concentrated products. The whole results indicated that 61% of the total aroma compounds were retained in the juice concentrated by OD; on the other hand, 74% of aroma compounds were lost during thermal evaporation. Phenolic compounds were also better preserved in the OD process: a 16% reduction of phenolic compounds was observed in the thermally evaporated juice in comparison to the raw juice [44].

According to the economic analyses, the profit of the OD process resulted lower than the evaporation process due to its higher equipment cost; however, the break-even point of the OD process was higher than that of the evaporation process indicating a greater vulnerability of the OD process to the variations in the production and sale. Typical applications of OD in pomegranate juice concentration are summarized in Table 4.

## 7. Aroma Recovery

Conventional processes of aroma recovery from agro-food products include solvent extraction, flash distillation and adsorption. However, these processes present some drawbacks such as limited recovery efficiencies and yields, due to the reactivity and low stability of aroma compounds, as well as high energy consumption.

The use of PV is a promising tool for the recovery of aroma compounds since it can be operated at moderate temperatures; moreover, the lack of chemical agents in the extraction process contributes positively to the preservation of the flavour and aroma compounds’ functional properties, minimizing the risk of possible contamination [110,111].

Raisi et al. [112] investigated the effects of key parameters on the pervaporative recovery of volatile aroma compounds from pomegranate juice with flat-sheet membranes in polydimethylsiloxane (PDMS) and polyoctylmethylsiloxane (POMS) with different thickness. Feed flow rate was shown to have no significant effect on both total flux and aroma enrichment factor in the range of 0.24–6.06 L min^−1^ (corresponding to Reynolds numbers between 100 and 2500). Thus, the effect of concentration polarization was considered negligible. On the other hand, an increase in feed temperature increased the diffusion rate of individual permeating molecules leading to high permeation fluxes and enrichment factors.

Total flux decreased by increasing the permeate pressure; for some aroma compounds, such as isopentyl acetate and α-ionone, the effect of permeate pressure on the flux was almost negligible while 3-methyl butanal and n-hexanol fluxes decreases with permeate pressure.

The selected membranes yielded higher enrichment factors on 3-methyl butanal and isopentyl acetate than on n-hexanol and α-ionone. This phenomenon was attributed to the reduced activity coefficient of organics in water by the hydroxyl group in the alcohol and the larger molecular size of α-ionone and to its low diffusion rate in the membrane. Higher total fluxes and lower enrichment factors were observed for PDMS membranes in comparison to the POMS membranes.

**Figure 7:**
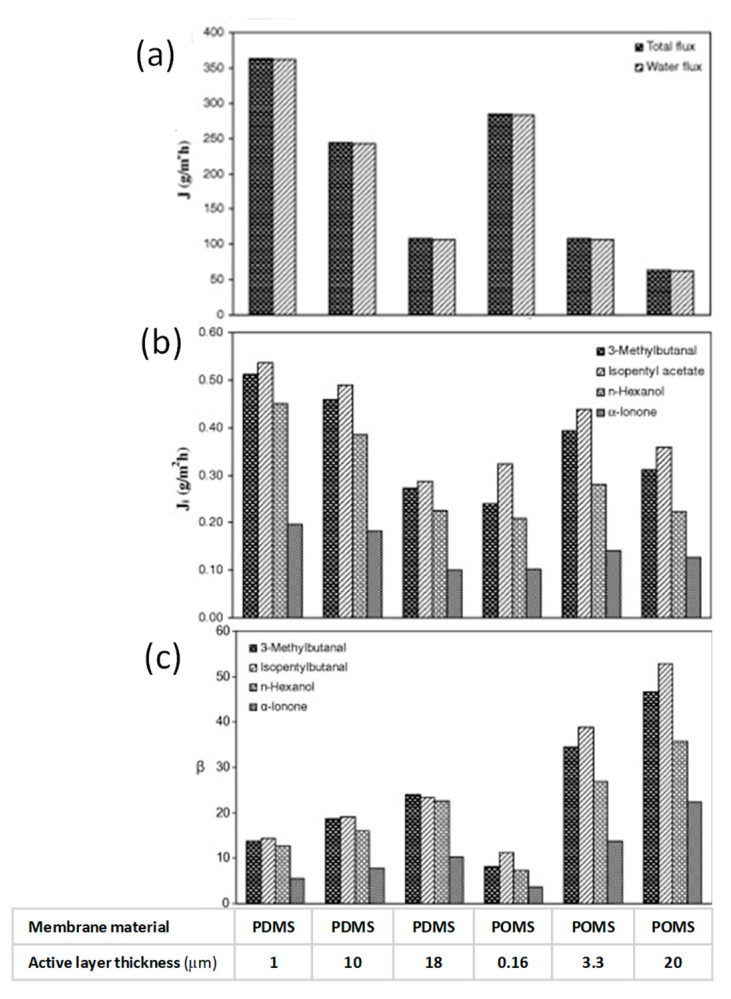
Effect of membrane active layer thickness on total and water flux (**a**), aroma compound fluxes (**b**) and aroma compound enrichment factors (**c**) of PDMS and POMS membranes for pervaporation of pomegranate aroma compounds from their binary feed solutions (adapted from [114]).

Raisi et al. [113] developed also a mass transfer model to predict the permeation flux and selectivity of aroma compounds through the PDMS membrane based on the solution-diffusion model. The model enables to describe the effects of feed concentration and feed temperature on the aroma compounds fluxes and selectivity. The predicted fluxes and selectivities were in agreement with experimental data. The predicted and experimental permeation fluxes increased by increasing temperature and aroma concentration in the feed solution. Authors evaluated also the effect of membrane thickness on the permeate flux and selectivity with binary and ternary solutions of the pomegranate aroma compounds in selected operating conditions (feed temperature 30 °C, permeate pressure 1 mmHg, feed flow rate 5 L min^−1^) [114]. The results showed that total and aroma permeation fluxes increased with a decrease in thickness of the selective layer; on the other hand, enrichment factors resulted significantly greater for the thicker membranes (Figure 7). In addition, the permeation of some aroma compounds was affected by presence of other aroma compounds.

## 8. Conclusions

Membrane-based operations implemented in pomegranate juice processing have been reviewed in the light of their growing interest as methodologies to preserve the overall quality of the juice. Microfiltration and ultrafiltration have been proven to be comparable to pasteurization in guarantee the microbiological stability of the juice, avoiding spoilage of the final product. Juice freshness, aroma and nutritional value are well preserved in comparison to the use of fining agents allowing to obtain high-quality, natural fresh-tasting and additive-free clarified products. Tight ultrafiltration and nanofiltration membranes offer new perspectives in juice fractionation aimed at recovering and purifying bioactive compounds of interest for the production of functional ingredients. Reverse osmosis and osmotic distillation represent useful alternatives to thermal evaporation for juice concentration. High levels of total soluble solids can be reached through osmotic distillation operating at low pressure and temperatures so avoiding mechanical and thermal stress of the processed juice.

The overall results indicate that the process is very efficient in preserving the original characteristics of the fresh juice including colour, total antioxidant activity, phenolic compounds and organic acids.

Pervaporation is a developing alternative technology appropriate for the concentration of aroma compounds from the juice due to the efficient and economical feature.

All these technologies represent also a useful approach for processing juice of underused fruits wasted as by-products generating more profits and minimizing production losses along with the subsequent environmental benefits. Finally, the integration of different membrane operations among themselves or with other conventional technologies represents today an emerging approach to redesigning the conventional flow diagram of pomegranate juice production with significant advantages in terms of product quality, recovery of health-promoting compounds, reduction of energy consumption and environmental impact.

## Figures and Tables

**Table 1:** Pomegranate juice composition from three varieties (Wonderful, Chaca and Codpa) at full ripe stage (adapted from [17]).

Parameter		Variety	
	Wonderful	Chaca	Codpa
pH	3.61 ± 0.01	3.68 ± 0.02	3.66 ± 0.02
TSS, °Brix	18.7 ± 0.3	18.4 ± 0.1	18.0 ± 0.1
TTA, % citric acid	1.04 ± 0.01	0.92 ± 0.01	1.04 ± 0.01
Ratio, L juice kg^−1^ arils	0.56	0.59	0.60
TPC, (mg GAE 100 L^−1^)	1930 ± 20	2100 ± 50	1600 ± 70
Antioxidant activity, (mmol trolox equivalent L^−1^)	11.5 ± 0.3	12.2 ± 0.3	14.9 ± 0.05
Delphinidin 3,5-diglucoside (mg Mv3 100 mL^−1^)	13.5 ± 1.2	11.7 ± 1.0	17.5 ± 1.2
Cyanidin 3,5-diglucoside (mg Mv3 100 mL^−1^)	36.4 ± 2.4	23.8 ± 0.7	34.7 ± 1.5
Pelargonidin 3,5-diglucoside (mg Mv3 100 mL^−1^)	0.7 ± 0.02	0.21 ± 0.02	1.9 ± 0.06
Delphinidin 3-glucoside (mg Mv3 100 mL^−1^)	1.4 ± 0.06	1.0 ± 0.04	2.5 ± 0.06
Cyanidin 3-glucoside (mg Mv3 100 mL^−1^)	10.6 ± 0.9	3.0 ± 0.4	11.8 ± 1.3
Pelargonidin 3-glucoside (mg Mv3 100 mL^−1^)	0.3 ± 0.02	0.2 ± 0.01	0.4 ± 0.002
Total anthocyanin content (mg Mv3 100 mL^−1^)	62.8 ± 3.7	43 ± 0.6	68.7 ± 1.0

TSS, total soluble solids; TTA, total titratable acidity; TPC, total phenolic content; GAE, gallic acid equivalents; Mv3, Malvidin-3-glucoside.

**Table 2:** Pomegranate juice clarification by MF and UF membranes.

Process	Membrane Type	Operating Conditions	Reference
MF	Flat-sheet, PVDF, 0.22 μm and 0.45 μm, 0.0209 m^2^ (Millipore, Billerica, MA, USA)	TMP, 0.5, 2 and 5 bar; CFV, 0.095 and 0.531 m s^−1^	[51]
MF	Flat-sheet, MCE, 0.22 μm and 0.1 μm, 0.0209 m^2^ (Millipore, Billerica, MA, USA)		
UF	Flat-sheet, MCE, 0.025 μm, 0.0209 m^2^ (Millipore, Billerica, MA, USA)		
MF	Flat-sheet, PVDF, 0.22 μm and 0.45 μm, 137.5044 × 10^−4^ m^2^ (Millipore, Billerica, MA, USA)	TMP, 0.5 bar; T, 25 °C	[52]
MF	Flat-sheet, MCE, 0.1 μm, 0.0209 m^2^ (Millipore, Billerica, MA, USA)	TMP, 0.5 bar; CFV, 0.0957 and 0.526 m s^−1^	[53]
UF	Flat-sheet, CA, 10 kDa, (YM10, Amicon Co, Danvers, MA, USA)	TMP, 2 bar	[54]
MF	Flat-sheet, PVDF, 0.22 μm, (Millipore, Billerica, MA, USA)	Not reported	[55]
UF	Hollow fiber, PEEK-WC, Dextran 68,800 MW rejection 10%, 46 cm^2^ (prepared in laboratory)	TMP, 0.96 bar; Q_f_, 1166 mL min^−1^; T, 25 °C;	[56]
UF	Tubular, ZrO_2_-TiO_2_, 15 kDa, 75 cm^2^ (Carbosep M2, Orelis, Miribel, France)	TMP, 2 bar; CFV, 1 m s^−1^; T, 20 °C;	[57]
MF	Flat-sheet, MCE, 0.22 μm, 0.0209 m^2^ (Millipore, Billerica, MA, USA)	Not reported	[58]
UF	Flat-sheet, MCE, 0.025 μm, 0.0209 m^2^ (Millipore, Billerica, MA, USA)		
UF	Tubular ZrO_2_-TiO_2_, 15 kDa, 75 cm^2^ (Carbosep M2, Orelis, Miribel, France)	TMP, 2 bar; CFV, 1 m s^−1^	[59]
UF	Tubular, ZrO_2_-TiO_2_, 15 kDa, 75 cm^2^ (Carbosep, Orelis, Miribel, France)	TMP, 1–3 bar; Q_f_, 0.25-0.95 L/min; T, 20–40 °C	[60]
MF	Hollow fiber, PEEK-WC, 2500 kDa, 26.9 cm^2^ (prepared in laboratory)	TMP, 1.15 bar; Q_f_, 59 L/h; T, 25 °C	[61]
	Hollow fiber, PS, 2000 kDa, 32.3 cm^2^ (prepared in laboratory)	TMP, 1.55 bar; Q_f_, 68 L/h; T, 25 °C	
UF	Flat-sheet, PVDF, 30 kDa, 0.0155 m^2^ (JW, GE Osmonics, Minnetonka, MN, USA)	TMP, 3 bar; Q_f_, 700 L/h; T, 25 °C	[62]
MF	Flat-sheet, MCE, 0.22 μm, 78 cm^2^ (Millipore, Billerica, MA, USA)	TMP, 0.5 bar; 80.7–341.9 Re	[63]
MF	Flat-sheet, MCE, 0.22 μm, 0.013 m^2^ (Millipore, Billerica, MA, USA)	TMP, 0.5 bar	[64]
MF	Flat-sheet, MCE, 0.45 μm, 78 cm^2^ (Millipore, Billerica, MA, USA)	T, 32–50 °C	[65]
MF	Hollow fiber, PEEK-WC, 2500 kDa, 26.9 cm^2^ (prepared in laboratory)	TMP, 0.35–1.35 bar; CVF, 1.12-4.2 m s^−1^; T, 25 °C	[66]
	Hollow fiber, PS, 2000 kDa, 32.3 cm^2^ (prepared in laboratory)	TMP, 0.35–1.35 bar; CFV, 1.12–4.2 m s^−1^; T, 25 °C	
UF	Tubular, PVDF, 200 kDa, 0.864 m^2^ (XPI-201, ITT PCI Membrane Systems, Zelienople, PN, USA)	TMP, 3.8 bar; h; T, 25 °C	[8]
MF	Hollow fiber, PVDF, 0.13 μm, 27.6 cm^2^ (prepared in laboratory)	TMP, 0.6 bar; Q_f_, 30 L/h; T, 25 °C	[67]
MF	Hollow fiber, PS, 0.13 μm, 27.6 cm^2^ (prepared in laboratory)	TMP, 0.6 bar; Q_f_, 30 L/h; T, 25 °C	
UF	Hollow fiber, CTA, 150 kDa, 0.26 m^2^ (FUC 1582, Microdyn-Nadir, Wiesbaden, Germany)	TMP, 0.6 bar, Q_f_, 400 L/h; T, 25 °C	[68]
MF	Flat-sheet, MCE, 0.22 μm, 78 cm^2^ (Millipore, Billerica, MA, USA)	Not reported	[69]
MF	MCE, 0.45 µm	Not reported	[70]
MF	Hollow fiber, PVDF, 0.13 μm, 27.6 cm^2^ (prepared in laboratory)	TMP, 0.6 bar; Q_f_, 30 L/h; T, 25 °C	[71]

Legend: MF, microfiltration; UF, ultrafiltration; MCE, mixed-cellulose ester; CTA, cellulose triacetate; CA, cellulose acetate; PS, polysulphone; PES, polyethersulphone; PVDF, polyvinylidene fluoride; PEEK-WC, modified poly(ether ether ketone); TMP, transmembrane pressure; Q_f_, feed flowrate; CFV, crossflow velocity; T, temperature; Re, Reynolds number).

**Table 3:** Variations in chemical composition and main characteristics of pomegranate juice following clarification and different concentration processes [108].

	RJ	CJ	OD	OMD	TE
pH	3.38 ± 0.03	3.35 ± 0.01	3.36 ± 0.03	3.34 ± 0.02	3.35 ± 0.02
TTA, g/100 mL	1.12 ± 0.05	1.11 ± 0.02	1.12 ± 0.03	1.13 ± 0.02	1.12 ± 0.04
L*	26.2 ± 0.2	29.2 ± 0.1	29.0 ± 0.1	29.2 ± 0.1	29.0 ± 0.1
a*	14.4 ± 0.1	13.1 ± 0.1	13.2 ± 0.1	13.2 ± 0.1	11.1 ± 0.1
b*	2.21 ± 0.07	3.04 ± 0.04	2.99 ± 0.05	3.01 ± 0.03	3.03 ± 0.03
ΔE	–	3.34 ± 0.08	3.16 ± 0.04	3.26 ± 0.08	4.39 ± 0.06
Hue	8.53 ± 0.13	13.0 ± 0.4	12.4 ± 0.2	12.4 ± 0.2	15.1 ± 0.1
Chroma	14.6 ± 0.1	13.5 ± 0.1	13.6 ± 0.1	13.5 ± 0.1	11.5 ± 0.1
TPC, g/100 mL	296 ± 11	222 ± 5	222 ± 5	222 ± 8	222 ± 8
TEAC, mM	19.6 ± 0.2	16.5 ± 0.3	16.2 ± 0.5	16.6 ± 0.4	14.5 ± 0.5
TMA, mg/L	346 ± 5	294 ± 6	274 ± 6	280 ± 4	247 ± 5
HMF, mg/L	n.d.	n.d.	n.d.	n.d.	0.32 ± 0.04
Gallic acid, mg/L	326 ± 5	285 ± 7	280 ± 9	281 ± 6	297 ± 10
Ellagic acid, mg/L	130 ± 5	110 ± 9	112 ± 4	111 ± 5	165 ± 10
(+)-catechin, mg/L	100 ± 4	71.1 ± 5.4	68.3 ± 8.6	69.4 ± 7.6	67.3 ± 2.4
Chlorogenic acid, mg/L	52.9 ± 2.8	46.4 ± 4.6	44.7 ± 4.1	45.2 ± 2.1	42.9 ± 3.8
Caffeic acid, mg/L	6.25 ± 1.64	3.74 ± 0.67	3.54 ± 0.54	3.62 ± 0.75	3.25 ± 0.88
Ferulic acid, mg/L	1.95 ± 0.87	n.d.	n.d.	n.d.	n.d.
Citric acid, g/L	11.4 ± 0.6	11.4 ± 0.4	11.5 ± 0.6	11.5 ± 0.4	11.6 ± 0.3
Malic acid, g/L	0.55 ± 0.07	0.62 ± 0.04	0.53 ± 0.05	0.54 ± 0.04	0.58 ± 0.08
Quinic acid, g/L	0.25 ± 0.03	0.23 ± 0.04	0.25 ± 0.04	0.24 ± 0.02	0.23 ± 0.02
Oxalic acid, g/L	5.97 ± 1.43	5.95 ± 1.25	5.97 ± 1.23	5.96 ± 0.98	5.95 ± 1.02

Results were corrected for 17.1 ± 0.1 °Brix. Values are mean ±SD, *n* = 3. RJ, raw juice; CJ, clarified juice; OD, osmotic distillation; OMD, osmotic membrane distillation; TE, thermal evaporation; L*, lightness index; a*, redness; b*, yellowness; n.d., not determined.

**Table 4:** Concentration of pomegranate juice by osmotic distillation.

Feed	Osmotic Agent	Membrane Type	Average Flux (kg/m^2^h)	Reference
Clarified juice by UF	CaCl_2_x2H_2_O, 10.2 M	hollow fiber, PP (Liqui-Cel^®^ Extra-Flow 2.5 × 8-in. membrane contactor, Hoechst-Celanese, Wiesbaden, Germany)	0.5	[56]
Clarified juice by UF	CaCl_2_x2H_2_O, 65% w/w	hollow fiber, PP (MD 020 CP 2N, Microdyn-Nadir, Wiesbaden, Germany)	1.1	[108]
Clarified juice by UF	CaCl_2_, 6 M	hollow fiber, PP (Liqui Cel^®^ minimodule 1.7 × 5.5 in., Membrana, Charlotte, NC, USA)	0.62	[104]
Clarified juice by PP spun filter)	CaCl_2_x2H_2_O, 6 M	flat-sheet, PTFE and PVDF (TS Filter, Hangzhou, China)	0.7 (PVDF); 1.5 (PTFE)	[105]
Clarified juice by UF and preconcentrated by plasma modified RO membranes	CaCl_2_x2H_2_O, 65% w/w	capillary, PP (MD 020 CP 2N, Microdyn-Nadir, Wiesbaden, Germany)	0.65	[94]
Raw juice	CaCl_2_, 5.43 M	nanofibrous PEBA membrane on a non-woven polyester substrate (prepared in laboratory)	2-2.3	[44]
